# Supplementary material for: Navigational strategy dictates hippocampal representation of space in an everyday memory task
Source: Nat Commun. 2026 Sep 24;17:11070. doi: 10.1038/s41467-026-77885-3 (PMC13691088; doi:10.1038/s41467-026-77885-3)
Supplement: Supplementary file 2 [file 41467_2026_77885_MOESM2_ESM.pdf]

nature portfolio

Peer Review File

Navigational strategy dictates hippocampal representation of space in an everyday memory task

Corresponding Author: Dr Francesco Gobbo

This file contains all reviewer reports in order by version, followed by all author rebuttals in order by version.

Version 0:

Reviewer comments:

Reviewer #1

(Remarks to the Author)
This is an ambitious and well-designed study that compares allocentric and egocentric strategies in a sophisticated behavioral task, combining miniscopes and optogenetic interventions. The behavioral paradigm is robust, and the combination of calcium imaging with causal manipulations of CA1 activity provides valuable insights into the role of hippocampal representations during planning and navigation. The study is timely and potentially impactful. However, several technical and interpretational issues need to be addressed before the manuscript can be considered for publication. I therefore recommend major revision.
Major points:
1- Unless I missed it, the manuscript does not report the number of cells imaged per session/animal. This information is important because population-level analyses, such as place field mapping, CCA correlations, and reactivation precision, can be influenced by the size of the recorded ensemble. If only a limited subset of CA1 neurons is sampled (as suggested by Supplementary Fig. S4), this could potentially bias the quantification of place field counts, reactivation events, and trial-to-trial neural trajectory similarity. The authors should report these numbers explicitly and, if possible, assess how robust the results are to variations in ensemble size (e.g., by subsampling or bootstrapping).
2- Most calcium activity is movement-related, but here the analysis focuses on a 10 s stationary window in the start box. Given the slow kinetics of GCaMP, it could be beneficial to show evidence that there is sufficient signal to reliably capture reactivation events during this period. I would like to see representative raw traces to assess the quality and timing of these reactivations.
3- The authors report that reactivation events in egocentric-trained rats map to multiple sandwells, whereas allocentric-trained rats reactivate a single location. This is an interesting observation but could be confounded by differences in the number of active neurons or place cells between groups. Some control analyses (e.g., down-sampling the allocentric dataset to match cell counts) may be needed.
4- The viral expression and spread of JAWS are well demonstrated, but the role of SWRs in the observed behavioral deficits remains unclear. Ripple occurrence is abolished in both egocentric and allocentric animals during startbox inhibition, yet only allocentric performance is impaired. Are the pre-startbox reactivations associated with SWRs? If so, why would ripple suppression not affect egocentric planning? Alternatively, does CA1 inactivation impact allocentric performance through a mechanism independent of ripples? This requires additional analyses and clarification in both the Results and Discussion.
Other comments:
5- The comparison of place field properties (number, size, directionality) between strategies needs methodological validation. Were the same criteria and thresholds applied across groups, and how robust are the results to parameter changes? Also, since egocentric-trained animals have fewer place cells but more fields per cell, could this be a detection bias due to different activity levels? Additional controls or resampling analyses would strengthen this point.
6- The CCA analysis is powerful, but I am concerned about the interpretation. Could differences in trial-to-trial variability or baseline firing rates, rather than true structural differences in neural representations, account for the observed higher canonical correlations in allocentric animals? A clearer explanation of how CCA handles partially overlapping neuronal ensembles would help.
7- A broader discussion is needed on how differences in recorded cell numbers and GCaMP expression patterns might influence the interpretation of allocentric vs. egocentric representations. Supplementary Fig. S4 suggests a potential bias toward deep pyramidal cells, which are known to exhibit distinct place coding and population-level transformations compared to superficial cells, particularly when allocentric and egocentric cues are in conflict. Could such laminar differences partially explain the observed variations in field numbers and trial correlations between the two groups?

Addressing this limitation and placing the findings in the context of recent studies on depth-specific population representations would strengthen the manuscript.
8- The labeling order of $GCaMP^{+}$ and $GCaMP^{-}$ in S4b–d is reversed across panels, which can easily confuse the reader. I recommend standardizing the labeling order throughout the figure.

Reviewer #2

(Remarks to the Author)
This work used an event arena-like apparatus and demonstrated that, depending on the training protocol, animals may or may not require distal external cues for accurate navigation to fixed food reward locations; they define distal cue-based navigation as allocentric, while the other as egocentric navigation. Then they compared how often cells representing different maze locations are active in the start box in the allocentric and egocentric conditions, which they refer to as reactivation. Finally, they show that blocking activity in the startbox impairs distal cue-based navigation. Conceptually, and experimental design-wise, this "egocentric allocentric" discrimination task used here is similar to their previous work (Nonaka et al., 2017, European J Neuroscience). Furthermore, there are several conceptual and technical concerns related to the current work.

Major comments

It is unclear from the data whether animals with the fixed startbox task always use an egocentric strategy. Past work showed that animals may initially use an allocentric navigation strategy and they can generalize to a hippocampus-independent egocentric strategy over several days of training (pubmed.ncbi.nlm.nih.gov/8673408/). Consequently, any differences seen in imaging could be due to differences in the training protocol only. The fact that animals can perform the task in the absence of distal cues does not necessarily suggest egocentric navigation. Furthermore, the error rates in variable startbox groups simply reflect the fact that animals need to rely on distal cues for navigation in that condition, so naturally their performance is impaired when the curtain is closed. Navigation to the mirror location using the opposite arm is the only indication that animals at the time of that specific test may have used an egocentric strategy (provided that distal clues were available). But it is unclear when such tests were performed, if they were performed later during training, these could indicate the generalization from allocentric to egocentric representation. Also, it is not clear whether distal cues were available. The only real indication that animals use a hippocampus-independent egocentric strategy would be to show that complete blockade of the hippocampus does not lead to a reduction of task performance. Optogenetic experiments only accomplished partial disruption of hippocampal activity, which led to partial behavioral impairment even in the variable start box condition.

Spatial activity-related characterisation of the imaged neurons would require more detailed analysis to account for other possible explanations.. Moreover, in the literature, reactivation refers to the reactivated activity of neuronal populations, while here only single-cell activity was examined, which does not take into account the cooperative firing of cells, only examines their independent activity. In this regard, comparisons of the results with the literature are not valid because the literature examined network-wide effects, while here, single-cell effects were checked. Furthermore, about the results here: can it be excluded that cells firing at reward locations are not place cell activity, but cells firing in relation to hippocampal sharp waves that can occur as the animal stops to grab the rewards and naturally, we expect frequent sharp waves in the start box as well. Also, if a larger proportion of cells are active at the goal location, it is also expected that, by chance, more of these cells will also be active at the start box.

It is unclear to what degree the optogenetic effects are due to partial disruption of hippocampal place cells or hippocampal sharp waves. Previous work, supriningly not cited (Jadhav et al., 2012, Science) showed that SWR disruption leads to a specific deficit of outbound trials on the W-mase task, indicating a working memory deficit (which would also require local cue-based turning left-right decisions). In contrast, in that case, inbound trials requiring the use of distal cues (reference memory) did not lead to impairment. The discrepancy of these results should be explained.

Reviewer #3

(Remarks to the Author)
This work examines how different navigational strategies, egocentric and allocentric, influence hippocampal prospective neural representations and spatial memory reactivation. This is an important, unsolved question in the field. The authors recorded dorsal hippocampal Ca^{2+} activity with head-mounted miniscopes in behaving rats. Animals performed a spatial memory task in a 2D arena using either allocentric or egocentric navigation strategies. Neural ensemble activity during navigation on the maze and inter-trial periods in a home box were analyzed and compared across task types. The main conclusion is hippocampal neural reactivation of rewarded locations in the arena during inter-trial periods was stronger in allocentric compared to egocentric task sessions. In addition, optogenetic inhibition of the dorsal hippocampus during inter-trial periods degraded task performance in allocentric but not egocentric navigation conditions.
This study addresses a central problem in the field that has been notoriously difficult to solve. It uses a powerful and original behavioral paradigm and reports several interesting findings regarding animal behavioral strategies and the causal role (or lack thereof) of the hippocampus. However, the recording, analysis and interpretation of the physiological data, have major caveats. The framing of the study and interpretation of the results have major flaws and do not take into account well established phenomena in the field. Overall, the results presented do not support the authors' interpretation, and the conclusions of the study are misleading. In my opinion, these caveats need to be addressed, which would require a substantial rework of the manuscript, before this study can be published in this or any other journal. I therefore focused my

points below on these main issues.

1- Through the manuscript the framing and interpretation of the study conflates several distinct hippocampal physiological phenomena, leading to misleading conclusions. The abstract opens talking about memory “replay”, then the following Introduction mixed references about hippocampal memory replay and reactivation, with others about theta sequences and slower-timescale remote activity. These are all very different phenomena with distinct mechanisms and likely different functions. Crucially, memory replay, which is the phenomena the authors state their study investigates, involves the reactivation during “offline” periods (such as intern-trial intervals in the home box in this case) of compressed temporal sequences of place cells. Replay, as described numerous times, requires the coordination of hippocampal cells in a millisecond timescale. This is not what the data in this manuscript shows. Furthermore, it seems unlikely their recording method can even be used to detect replay in the first place, due to the slow temporal resolution of 1-photon Ca2+ imaging. Other types of hippocampal remote or prospective the manuscript alludes too, without clearly defining them, such as the reactivation of cell assemblies of theta sequences, are also based on millisecond-timescale organization of spiking patterns. This is again not what the authors measured. In my opinion, all the interpretation about replay and reactivation needs to be removed from the paper, otherwise it would be highly misleading for general readers.

2- The method employed to assess hippocampal “reactivation”, used to support some of the main claims of the paper (Fig 3-4) is not adequate. The authors should use some of the well well-established methods in the field, which are based on performing careful statistical testing of the significance of the temporal structure of neural activity during offline periods compared to neural patterns during behavior in the maze. Instead, they simply counted which cells were active in 10 s periods before trial start. This measure was them compared with manual (?) annotations of whether those cells were active when rats were exploring different sandwells. This approach is very far from current standards in the field and prevents any meaningful interpretation of the results of hippocampal activity during inter-trial periods. Among the many caveats, the approach does not consider the temporal structure of activity, it is biased by baseline activity levels of different cells, does not control for differences in brain state that correspond to very different network activity (e.g., theta oscillations and ripples), is not validated by proper statistical and shuffling methods.

3- The optogenetic manipulation results are useful and interesting. While the differential behavioral impact on allocentric and egocentric conditions is valuable, its underlying mechanisms are not possible to determine with the data presented. Furthermore, such prolonged silencing (3 min) is very problematic for multiple reasons. Among them, it silences different types of activity patterns (ripples as highlighted by the authors, but also theta oscillations and others), it allows time for effects to propagate through polysynaptic circuits beyond the hippocampus, can lead to cell accommodation and tissue over-heating. These caveats prevent a mechanistic interpretation of the behavioral effects. A better approach, nowadays common in the field, would be to transiently silence the hippocampus during specific activity patterns (such as ripples) or at least during specific discrete behavioral periods (perhaps harder in the context of this task).

Version 1:

Reviewer comments:

Reviewer #1

(Remarks to the Author)
I thank the authors for their thoughtful and extensive revisions, which have substantially improved the manuscript. The additional analyses, hierarchical bootstrap controls, decoder validation, and new figures documenting example Ca^{2+} traces and cell counts all strengthen the methodological rigor. The clarifications on place-field detection parameters, the reorganization of the text, and the updated discussion are also appreciated.

That said, several conceptual issues remain only partially resolved and would benefit from further clarification.

1. Nature of “reactivation” and temporal limitations of Ca^{2+} imaging: The authors now refrain from using the term replay, which is a helpful step. However, the interpretation of the pre-trial hippocampal activity still remains conceptually difficult. Even with decoders and automatic cell classification, the underlying measure is a coarse quantification of which neurons were active during a 10 s period—not a temporal sequence, and not an event with defined network structure. I appreciate the authors’ transparent recognition of this limitation, but the interpretation still appears stronger than what these signals can support. A clearer distinction is still needed between sequential “replay” phenomena, which require millisecond precision and fast-time structured patterns, and reactivation of coarsely overlapping populations, which is what can be extracted from 1-photon Ca^{2+} imaging.The authors need further sharpen the conceptual boundary in the Introduction and Discussion so that readers do not infer a level of temporal structure that cannot be resolved with the current method.

2. Interpretation of optogenetic manipulations: The authors nicely document the illumination times and discuss SWR-independent forms of prospective activity. These additions help. Still, the core interpretive issue remains unresolved because prolonged inhibition inevitably affects multiple network states and perhaps extra-hippocampal circuits, making difficult to attribute the behavioral effect to suppression of a specific hippocampal computation in the pre-startbox window. The manuscript would be strengthened by explicitly framing the optogenetic results as a state-dependent perturbation of hippocampal activity during the planning window, rather than as evidence for a mechanism tied to a specific physiological event class (e.g., SWRs).

3. Regarding my earlier point on potential laminar biases: I appreciate the authors' expanded discussion on deep vs. superficial CA1 pyramidal cell differences. However,the revised text relies primarily on ephys studies, which given its millisecond temporal resolution captures dynamics that are not directly comparable to those measured with one-photon calcium imaging. Because the present study interprets differences in place coding and reactivation using calcium signals, it would be more appropriate to place the results in the context of imaging-based studies that have quantified depth-dependent sampling biases and their impact on spatial tuning and stability (e.g., PMID: 27397517; PMID: 40015277). Incorporating these imaging perspectives would clarify how potential laminar biases may influence the results and would make the discussion more internally consistent with the methodological framework of the paper.

(Remarks on code availability)
n/a

Reviewer #2

(Remarks to the Author)
The revision has largely addressed concerns, and particularly the discussion now appropriately discusses the limitations of the work. I would recommend including a block diagram (or diagrams) that summarizes all the experimental details related to what is tested at different sessions, and in Figure 1c,d, mark the 180-degree rotation trials. It is also curucial to mention that after session 19, the rotation trials were performed in curtain open (I assume) which is a cruicial detial, which i asked about in my previous review.

(Remarks on code availability)

Reviewer #3

(Remarks to the Author)
In this revision, the authors have included a few new analysis and statistical controls as well as textual additions to the Introduction and Discussion. While I appreciate the new additions and, more so, the extensive amount of data already contained in this manuscript, the concerns I raised in my original revision still remain largely unsolved. I will not ask for additional experiments or analyses, since this paper is already massive. Instead, I will reiterate below the major concerns with the framing of this study, interpretation of the results and general analytical framework that, unfortunately, still prevent me to endorse its publication.
One of my main initial concerns was the framing of the current study and its finding in relation to hippocampal "replay" or "reactivation". These are very well-established concepts in the field that refer to the "offline" recurrence of neural population activity patterns (either sequences in the case of replay or more generally ensemble cofiring in the case of reactivation). The two defining features of these physiological events are 1) they involve coordinated population activity, and 2) they rely on the preservation of the temporal structure of such activity but in a compressed manner compared to its occurrence during behavior. None of these features are present in the events and phenomena study in this work. With this, I do not want to imply that there is no value in the present work or in the observations reported by the authors. However, the current framing is very misleading and, in my view, the interpretation of the data is not accurate, and at odds with well-established standards in the field.
Despite my insistence on this same issue in my original revision, the only thing the authors have done to address it is to add some text in Introduction and Discussion to describe replay and other phenomena in the literature. While this is useful, it misses the main point which was reframing their interpretation. I will provide just a few examples to illustrate this point, but there are many more in the manuscript.
- The first sentence of the abstract still frames the problem relating it to the concept of replay: "It still remains unclear how the brain replays stored neural information…"

- The authors keep insisting that reactivation is an appropriated term to describe their data and that it does not pose any confound with prior literature. As a proof that this is not the case, they themselves use this same term to refer to their observed phenomena (essential the firing of individual place cells encoding specific maze locations during inter-trial intervals in the start box) with the replay of compressed place cell sequences or the reactivation of cell assemblies (e.g., lines 77-85, lines 567-573). I want to stress that this framework is very misleading. Unfortunately, the new text added to the revised manuscript sometimes increases the confusion because it compares the observation of the authors with other types of events previously studied (replay, assemblies or theta sequences) that are completely different.

A major shortcoming of the study is that the analytical framework is based in the analysis of the activation of single neurons, rather than in the temporal structure of population activity. While that is something the field has already moved pass from, if the authors still want to focus on this approach is wrong in itself. However, this should be clearly stated (currently it is not clearly evident) the interpretation of the data should be changed accordingly, and the comparison with prior studies of population patterns should be carefully made (currently it is not). For example, the paper (starting from the paper) keeps referring to the reactivation of planned spatial trajectories. This is far from accurate because reactivation of trajectories implies by definition a sequence of place cell. Instead, what the authors show is the "reactivation" of specific locations in the maze, not trajectories towards them. Again, the current framing and interpretation does a great disservice to the present work, which includes many interesting observations, and precludes an accurate understanding by readers.

The new analysis included, while very limited in scope, are helpful. It was not easy for me to track the new analysis and

figures since very little of them has been added to the main figures and text, and nothing is specifically labelled in the supplemental material. Overall it seems that the new analytical addition have been limited to provide some restricted controls while the main analysis framework, which was the object of my initial criticism, remains unchanged.

(Remarks on code availability)
NA

Version 2:

Reviewer comments:

Reviewer #1

(Remarks to the Author)
The authors have addressed the remaining concerns and I found the paper suitable for publication. Congrats on a very interesting study which will help to advance the field.

(Remarks on code availability)

Reviewer #3

(Remarks to the Author)
In this revision, the authors have thoroughly addressed the main remaining issue I previously raised. This concerned the framing of the study and interpretation of their results in relation with the phenomena of hippocampal replay/ reactivation. The authors have corrected previously confusing terminology and re-interpreted their findings in a way that more accurately reflects the data presented. I believe these changes will help readers better appreciate the contributions of this study . I do not have further points to raise.

(Remarks on code availability)

Open Access This Peer Review File is licensed under a Creative Commons Attribution 4.0 International License, which permits use, sharing, adaptation, distribution and reproduction in any medium or format, as long as you give appropriate credit to the original author(s) and the source, provide a link to the Creative Commons license, and indicate if changes were made.
In cases where reviewers are anonymous, credit should be given to 'Anonymous Referee' and the source.
The images or other third party material in this Peer Review File are included in the article's Creative Commons license, unless indicated otherwise in a credit line to the material. If material is not included in the article's Creative Commons license and your intended use is not permitted by statutory regulation or exceeds the permitted use, you will need to obtain permission directly from the copyright holder.

To view a copy of this license, visit https://creativecommons.org/licenses/by/4.0/

We thank the reviewers for their constructive criticism. We have embedded our responses in blue below.

REVIEWER COMMENTS

Reviewer #1 (Remarks to the Author): This is an ambitious and well-designed study that compares allocentric and egocentric strategies in a sophisticated behavioral task, combining miniscopes and optogenetic interventions. The behavioral paradigm is robust, and the combination of calcium imaging with causal manipulations of CA1 activity provides valuable insights into the role of hippocampal representations during planning and navigation. The study is timely and potentially impactful. However, several technical and interpretational issues need to be addressed before the manuscript can be considered for publication. I therefore recommend major revision.

Major points:

1- Unless I missed it, the manuscript does not report the number of cells imaged per session/animal. This information is important because population-level analyses, such as place field mapping, CCA correlations, and reactivation precision, can be influenced by the size of the recorded ensemble. If only a limited subset of CA1 neurons is sampled (as suggested by Supplementary Fig. S4), this could potentially bias the quantification of place field counts, reactivation events, and trial-to-trial neural trajectory similarity. The authors should report these numbers explicitly and, if possible, assess how robust the results are to variations in ensemble size (e.g., by subsampling or bootstrapping).

We thank the Reviewer for the comment and have plotted the number of cells in new Supplementary Figure S7, as well as the average number of cells detected in each session for each animal. We found that the vast majority (87%) of pyramidal neurons express GCaMP7f from histological analysis (Supplementary Figure S4) and think the main reason for uneven number of detected cells lies in the clarity of the field of view and relative orientation of the lens with respect to the curvature of the CA1 sub-field in the rat, which is particularly pronounced compared to mice. Most of our analyses are run with linear mixed models that include animals as random factors and therefore include a within-animal effect that minimises the effect of animal fixed features such as the number of cells. Furthermore, for reactivation events, the statistical analysis is run on trials with linear models, so while the number of cells can affect the precision of the value estimated for a given trial (e.g. fraction of SW1 reactivations), it should have minimal effect on the statistical distribution. Nevertheless, to exclude biases due to an uneven number of cells between animals and groups, we have also recalculated the parameters related to Place Field Properties (Proportion of Place Fields, Number of Place Fields and Directionality Index in Main Figure 2, and Place Field area and Stability in Supplementary Figures S11 and S13) with Hierarchical Bootstrap analysis (Saravanan et al, Neuron Behav Data Anal Theory 2020). This allows to estimate these parameters as extrapolated or resampled to an equal number of cells per animal, removing any effects due to e.g. a large number of samples from a single animal distorting results. The results obtained with Bootstrap analysis confirm our initial analysis performed with linear models on the measured data. We also provide estimates of how P values behave as a function of number of cells in the new Supplementary Figure S10.

2- Most calcium activity is movement-related, but here the analysis focuses on a 10 s stationary window in the start box. Given the slow kinetics of GCaMP, it could be beneficial to show

evidence that there is sufficient signal to reliably capture reactivation events during this period. I would like to see representative raw traces to assess the quality and timing of these reactivations.

The Reviewer is correct that traces have a slow kinetics, and that is the reason why we have used the events in our analysis. Event detection with CaImAn/OASIS results in the time stamp of the event to be at the maximum of the trace and hence within 1 or 2 temporal frames from trace onset. Most GCaMP variants, including the fast GCaMP6f used in this study, have a rapid rise time followed by a slower decay to baseline, which gives the trace its typical shape. We have now included several representative traces from animals recorded in both allocentric and egocentric tasks in the new Supplementary Figures S26-27 showing robust traces at various time points before the trial start (shaded area in the figures). In many instances, those corresponded to cells mapping to positions in the arena later traversed by the animals, as expected, showing comparable amplitudes. In some instances, those only mapped "potential" visits, consistent with our interpretation of possibility planning. As a note, while the Reviewer is correct that the animals are largely motionless in the startbox during those 10s (and further before), they usually display small movements in place such as head turning, as also shown in our work in PNAS (Gobbo et al PNAS 2022) and, occasionally, movement within the startbox.

3- The authors report that reactivation events in egocentric-trained rats map to multiple sandwells, whereas allocentric-trained rats reactivate a single location. This is an interesting observation but could be confounded by differences in the number of active neurons or place cells between groups. Some control analyses (e.g., down-sampling the allocentric dataset to match cell counts) may be needed.

We thank the Reviewer for the comment. We have plotted the number of cells in new Supplementary Figure S7 in the allocentric and egocentric case and found no consistent trend between animals that would indicate a larger number of cells active in one of the two groups. Furthermore, we plotted the number and the average event rate for cells mapping the various sandwells and were also active in the startbox (new Supplementary Figure S25) and found no significant difference between the two groups. We also recalculated the parameters related to Place Field Properties (Proportion of Place Fields, Number of Place Fields and Directionality Index in Main Figure 2, and Place Field area in Supplementary S10) with Hierarchical Bootstrap analysis to re-estimate these parameters for an equal number of cells per animal.

4- The viral expression and spread of JAWS are well demonstrated, but the role of SWRs in the observed behavioral deficits remains unclear. Ripple occurrence is abolished in both egocentric and allocentric animals during startbox inhibition, yet only allocentric performance is impaired. Are the pre-startbox reactivations associated with SWRs? If so, why would ripple suppression not affect egocentric planning? Alternatively, does CA1 inactivation impact allocentric performance through a mechanism independent of ripples? This requires additional analyses and clarification in both the Results and Discussion.

This is an interesting point that would require ad hoc experiments to address fully. For practical reasons, we did not record the field potential in the hippocampus along with miniscope recording. This would be necessary to see if events occur in association with SWRs. That would be difficult to implement, as in the few instances where field has been recorded along with

miniscopes, it was recorded from the opposite hemisphere (e.g. Kim e al Exp Neurobiol 2022). Furthermore, the temporal matching between LFP and events would be hard to estimate, given the temporal dynamics of calcium indicators. In animals implanted with optrodes, we found that JAWS stimulation significantly inhibited neural activity, and furthermore reduced the number of SWRs. It must be noted that reactivation events in general do not necessary occur only within SWR periods. For instance, Widlowski and Foster (Neuron 2022) report that "many replay-like events during immobility periods were unaccompanied by large ripples or population bursts".

Our finding was that JAWS stimulation results in a selective impairment in allocentric but not egocentric performance. This striking result is consistent with other reports showing that egocentric activity is insensitive to hippocampal pharmacological inactivation, but that allocentric activity is not (Packard and McGaugh, Neurobiology of Learning and Memory 1996). One possibility is that this planning relies on striatal activity. One possibility is that egocentric planning relies on striatal activity. This does not imply that some level of representation still occurs within the hippocampus, as also our data indicate, but that this activity is not necessary to solve the task. Interestingly, the striatal activity seems to have a dominant effect on allocentric representation, hence possibly on hippocampal activity, since striatal inhibition shift the behaviour of animals in the T/X-maze from response (egocentric) to place (allocentric) (Packard and McGaugh, Neurobiology of Learning and Memory 1996). These points have now been addressed in the Discussion.

Other comments: 5- The comparison of place field properties (number, size, directionality) between strategies needs methodological validation. Were the same criteria and thresholds applied across groups, and how robust are the results to parameter changes? Also, since egocentric-trained animals have fewer place cells but more fields per cell, could this be a detection bias due to different activity levels? Additional controls or resampling analyses would strengthen this point.

We thank the Reviewer for pointing this out; we have we recalculated the parameters related to Place Field Properties (Proportion of Place Fields, Number of Place Fields and Directionality Index in Main Figure 2, and Place Field area in Supplementary Figures S11,13) with Hierarchical Bootstrap analysis to re-estimate these parameters for an equal number of cells per animal. The results obtained with Bootstrap analysis confirm our initial analysis with linear models on the measured data. We also provide estimates of how P values behave as a function of number of cells in the new Supplementary Figure S10. Cells in the Exploration phase do not show significant differences in activity that could explain the higher number of fields (new Supplementary Figure S25).

We have applied the same criteria between egocentric and allocentric groups and run the classification by an automatic algorithm. We tested different parameters, such as varying the bin size, threshold for mutual information, number of traversals in the place field, and always found the trend was conserved between allocentric and egocentric groups for a given set of parameters. We think that the main reason for the difference is lower Mutual Information values, as our definition of Place Cell is base on MI vs chance. This could be due to extrafield activity or multiple place fields. Indeed, we found that cells in the egocentric group occasionally displayed fields with 2- or 4-fold rotational symmetry (e.g. mapping to the four starboxes doors, or arena corners) – event if we didn't inquire this further. We also have plotted the trend of p

value significance as the number of cells varies and found our data lie in a robust interval (new Supplementary Figure S10).

6- The CCA analysis is powerful, but I am concerned about the interpretation. Could differences in trial-to-trial variability or baseline firing rates, rather than true structural differences in neural representations, account for the observed higher canonical correlations in allocentric animals? A clearer explanation of how CCA handles partially overlapping neuronal ensembles would help.

The Reviewer raises a good point. Indeed, to account for uneven number of shared neurons between trials, and incorrect trials following unique trajectories, we had to exclude from this analysis a number of trials. The trials we included had similar trial-to-trial variability and number of active neurons between groups (Supplementary Figure S31). Because we thought our CCA analysis was of interest and complementary but not necessary to support our main findings, that are related to the predictive and planning role of the hippocampus between the two tasks, we also included a Supplementary Discussion addressing the points raised by the Reviewer and clarifying how various parameters could affect the CCA results in the Supplementary Material.

7- A broader discussion is needed on how differences in recorded cell numbers and GCaMP expression patterns might influence the interpretation of allocentric vs. egocentric representations. Supplementary Fig. S4 suggests a potential bias toward deep pyramidal cells, which are known to exhibit distinct place coding and population-level transformations compared to superficial cells, particularly when allocentric and egocentric cues are in conflict. Could such laminar differences partially explain the observed variations in field numbers and trial correlations between the two groups? Addressing this limitation and placing the findings in the context of recent studies on depth-specific population representations would strengthen the manuscript.

The Reviewer raises an interesting point. We likely recorded from a mixed population across the layers in the pyramidal layer but nevertheless it is impossible to know for sure. We have addressed this point in Discussion, including how neuronal position could affect the interpretation of our results.

8- The labeling order of $GCaMP^+$ and $GCaMP^-$ in S4b–d is reversed across panels, which can easily confuse the reader. I recommend standardizing the labeling order throughout the figure.

We thank the Reviewer for noticing; the order has been now harmonised in the Figure.

Reviewer #2 (Remarks to the Author): This work used an event arena-like apparatus and demonstrated that, depending on the training protocol, animals may or may not require distal external cues for accurate navigation to fixed food reward locations; they define distal cue-based navigation as allocentric, while the other as egocentric navigation. Then they compared how often cells representing different maze locations are active in the start box in the allocentric and egocentric conditions, which they refer to as reactivation. Finally, they show that blocking activity in the startbox impairs distal cue-based navigation. Conceptually, and experimental design-wise, this "egocentric allocentric" discrimination task used here is similar to their previous work (Nonaka et al., 2017, European J Neuroscience). Furthermore, there are several conceptual and technical concerns related to the current work.

Major comments It is unclear from the data whether animals with the fixed startbox task always use an egocentric strategy. Past work showed that animals may initially use an allocentric navigation strategy and they can generalize to a hippocampus-independent egocentric strategy over several days of training (pubmed.ncbi.nlm.nih.gov/8673408/). Consequently, any differences seen in imaging could be due to differences in the training protocol only. The fact that animals can perform the task in the absence of distal cues does not necessarily suggest egocentric navigation. Furthermore, the error rates in variable startbox groups simply reflect the fact that animals need to rely on distal cues for navigation in that condition, so naturally their performance is impaired when the curtain is closed. Navigation to the mirror location using the opposite arm is the only indication that animals at the time of that specific test may have used an egocentric strategy (provided that distal clues were available). But it is unclear when such tests were performed, if they were performed later during training, these could indicate the generalization from allocentric to egocentric representation. Also, it is not clear whether distal cues were available. The only real indication that animals use a hippocampus-independent egocentric strategy would be to show that complete blockade of the hippocampus does not lead to a reduction of task performance. Optogenetic experiments only accomplished partial disruption of hippocampal activity, which led to partial behavioral impairment even in the variable start box condition.

We agree with the Reviewer that animals do not necessarily follow an egocentric strategy at all points during the training – particularly at the beginning. As the Reviewer notes, rats develop the use of egocentric strategies over time (Packard and McGaugh, Neurobiology of Learning and Memory 1996). This is exactly why we trained the animals for an extensive number of session (27) prior to starting the recordings. By then, we could be, to the best of our ability, sure that animals were performing one task using allocentric coordinates, and the other by egocentric coordinates. Indeed, we aimed for differences to be due to the training protocol only in order to compare changes in representation between the two strategy used. Our aim was therefore to develop two distinct procedures that could produce one or the other navigational strategy as effectively as possible in a naturalistic way. Indeed, the description of the allocentric task, and the description of controls and alternative task structures, was reported in Broadbent et al (EJN, 2020). We had originally trained (and recorded) a separate group of animals in an egocentric version of the task simply by letting them start each session always from the same startbox. They showed convincing egocentric hallmarks, including capability of solving the task in absence of cues and higher number of errors when starting a test trial from a startbox different than the one used in Sample (learning trials). In the final protocol used we further induced the use of an egocentric strategy by training animals for the first 15 sessions in complete absence of cues – hence they could rely only on egocentric coordinates. We ran a number of controls: for allocentric animals, (i) in a choice trial, starting from a startbox different from those used in the

Sample/learning trials (this was actually the case in most sessions throughout training, but particularly in Sessions 8, 16, 24 and 32) – no difference was found, as expected; (ii) use of curtains in a control choice trial where all cues where removed (session 18,25,34) – as expected, performance was at chance; for egocentric animals: (i) use of curtains in a control choice trial where all cues where removed (session 18,25,34) – performance was not different than trials with cues available, suggesting they were not relying on them; (ii) use of 180 degree rotations of the starting point (sessions 8,16,24,32) – resulting in the identification of the correct sandwell in egocentric, but not allocentric, coordinates. We believe our controls are reasonable enough to demonstrate the use of one or the other set of coordinates, and that they are sparse enough along the whole training procedure to ensure that animals are performing consistently, at the very least during the sessions where neuronal activity was recorded with miniscopes: as shown in Figure 1, we recorded sessions 28 to 34, flanked by controls on either side of this sessions interval. The results of controls are reported in Figure 1 and we also included the results from allocentric control (i) (in a choice trial, starting from a startbox different from those used in the Sample) in Supplementary Figures 1 and 2.

Spatial activity-related characterisation of the imaged neurons would require more detailed analysis to account for other possible explanations. Moreover, in the literature, reactivation refers to the reactivated activity of neuronal populations, while here only single-cell activity was examined, which does not take into account the cooperative firing of cells, only examines their independent activity. In this regard, comparisons of the results with the literature are not valid because the literature examined network-wide effects, while here, single-cell effects were checked. Furthermore, about the results here: can it be excluded that cells firing at reward locations are not place cell activity, but cells firing in relation to hippocampal sharp waves that can occur as the animal stops to grab the rewards and naturally, we expect frequent sharp waves in the start box as well.

We agree with the Reviewer that there may be indeed discrepancies due to single cell vs population analysis. For this reason, we have avoided the term “replay” that may imply the recognition of a sequence of cells, or otherwise an ensemble of cells, and is already relatively well-defined in the literature. However, because nevertheless the information content of cells active in the arena is indeed re-activated in the startbox, we have used this term (“reactivation”) to indicate the phenomenon, as also already introduced in our previous paper (Gobbo et al PNAS 2022). Unfortunately, there are a limited number of words that can indicate this phenomenon. Nevertheless, we appreciate that reactivation can be used to indicate cooperative firing (e.g. Dupret et al Nat Neurosci 2010, Sugden et al Nat Neurosci 2020). We have therefore discussed the difference between population reactivation and our use of the term in the discussion. We believe this is a reasonable compromise and that the distinction should be sufficiently clear to the readers.

Indeed, we found that several cells with similar information content, such as cells mapping the same sandwell, are often activated within short periods of time (see for instance Figure 4), which could link our observation with population reactivation. However, the limitation of one-photon calcium imaging in terms of sensitivity and temporal resolution prevents us from inquiring about this further. A similar consideration prevents us from correlating the activity with sharp waves; we defined place maps using only points where the animal is moving, so the impact of reward-grabbing and consumption should be minimal, also considering that in the Exploration phase animals receive no pellets at sandwells. Equally, in the startbox, we

considered the last 10s before the start of the trial to make sure that any pellet retrieved in the arena in the previous trial or as cue pellet had been eaten. We have addressed this point in the Discussion. Last, our references to population reactivations are not meant to provide a direct comparison but to acknowledge existing data and their interpretation; we believe further experiments will clarify the relative contributions of information access by the hippocampus during trajectory or destination planning.

Also, if a larger proportion of cells are active at the goal location, it is also expected that, by chance, more of these cells will also be active at the start box.

The Reviewer raises a valid point. We have now listed the cells mapping onto a sandwell from the activity recorded in the Exploration and divided them by whether they were active at the goal location or at one of the other sandwells. We found no difference in the number of cells, or their percentage, that would explain this alternative hypothesis. The data is presented in new Supplementary Figure S25f.

It is unclear to what degree the optogenetic effects are due to partial disruption of hippocampal place cells or hippocampal sharp waves. Previous work, suprisingly not cited (Jadhav et al., 2012, Science) showed that SWR disruption leads to a specific deficit of outbound trials on the W-mase task, indicating a working memory deficit (which would also require local cue-based turning left-right decisions). In contrast, in that case, inbound trials requiring the use of distal cues (reference memory) did not lead to impairment. The discrepancy of these results should be explained.

We thank the Reviewer for pointing out this important work, that we have now included among our references as an example of live perturbation of hippocampal activity. While the task is structurally very different from ours, we would suggest that the discrepancy arises because in the W alternation maze task animals may still rely on allocentric coordinates in the outbound trials. As the Reviewer argues, there is a working memory component but that also requires cues to identify left and right. It is a situation not too dissimilar to the T-maze described in Packard and McGaugh, Neurobiology of Learning and Memory 1996, where the use of a 4th arm reveals that the choice in the first week is still based on the reliance on allocentric coordinates. Indeed, consistently with Jadhav et al, pharmacological hippocampal inactivation results in chance performance. As for inbound trials, while animals can of course use distal cues, alternative possibilities can emerge – and indeed could be even amplified by the experimental manipulation, such as choosing the first available arm (as no other arm is possible in the other direction), or tracing back to the previous arm by path integration, none of whose strategy would rely on the hippocampus.

Reviewer #3 (Remarks to the Author): This work examines how different navigational strategies, egocentric and allocentric, influence hippocampal prospective neural representations and spatial memory reactivation. This is an important, unsolved question in the field. The authors recorded dorsal hippocampal Ca2+ activity with head-mounted miniscopes in behaving rats. Animals performed a spatial memory task in a 2D arena using either allocentric or egocentric navigation strategies. Neural ensemble activity during navigation on the maze and inter-trial periods in a home box were analyzed and compared across task types. The main conclusion is hippocampal neural reactivation of rewarded locations in the arena during inter-trial periods was stronger in allocentric compared to egocentric task sessions. In addition, optogenetic inhibition of the dorsal hippocampus during inter-trial periods degraded task performance in allocentric but not egocentric navigation conditions. This study addresses a central problem in the field that has been notoriously difficult to solve. It uses a powerful and original behavioral paradigm and reports several interesting findings regarding animal behavioral strategies and the causal role (or lack thereof) of the hippocampus. However, the recording, analysis and interpretation of the physiological data, have major caveats. The framing of the study and interpretation of the results have major flaws and do not take into account well established phenomena in the field. Overall, the results presented do not support the authors' interpretation, and the conclusions of the study are misleading. In my opinion, these caveats need to be addressed, which would require a substantial rework of the manuscript, before this study can be published in this or any other journal. I therefore focused my points below on these main issues.

We thank the Reviewer for recognizing the study as addressing a 'central problem' in the field, appreciating our behavioural paradigm as 'powerful and original' and referring to our experimental findings as 'interesting'.

1- Through the manuscript, the framing and interpretation of the study conflates several distinct hippocampal physiological phenomena, leading to misleading conclusions. The abstract opens talking about memory "replay", then the following Introduction mixed references about hippocampal memory replay and reactivation, with others about theta sequences and slower-timescale remote activity. These are all very different phenomena with distinct mechanisms and likely different functions. Crucially, memory replay, which is the phenomena the authors state their study investigates, involves the reactivation during "offline" periods (such as intern-trial intervals in the home box in this case) of compressed temporal sequences of place cells. Replay, as described numerous times, requires the coordination of hippocampal cells in a millisecond timescale. This is not what the data in this manuscript shows. Furthermore, it seems unlikely their recording method can even be used to detect replay in the first place, due to the slow temporal resolution of 1-photon Ca2+ imaging. Other types of hippocampal remote or prospective the manuscript alludes too, without clearly defining them, such as the reactivation of cell assemblies of theta sequences, are also based on millisecond-timescale organization of spiking patterns. This is again not what the authors measured. In my opinion, all the interpretation about replay and reactivation needs to be removed from the paper, otherwise it would be highly misleading for general readers.

We thank the Reviewer's for this insight. Our aim is to investigate what the hippocampal activity looks like in the startbox and in general during memory-based decision making when animals are solving a task relying on allocentric coordinates or otherwise on egocentric coordinates. The overarching interest of the question is what happens in the hippocampus when its activity is not

strictly necessary to solve the task, as it could be inferred by the available literature, and confirmed in our optogenetics experiments. For this reason, we have avoided the term “replay” in describing the activity that we record in the startbox, which would have implied the recognition of a sequence of cells, or otherwise an ensemble of cells, and is already relatively well-defined in the literature. Instead, we have used the term reactivation, as also previously in Gobbo et al PNAS 2022, to avoid confusion. Furthermore, we absolutely agree with the Reviewer that the technique employed in our work does not have the necessary temporal resolution and sensitivity to capture and describe phenomena acting on the tens-of-millisecond time scale such as replay. On the other hand, it allows a trustworthy identification of neurons across days, allowing distinct comparisons that are not possible with electrophysiology. This was discussed transparently in our previous paper and we have now discussed it further in the Discussion for clarity. Nevertheless, it would be a limitation of the study to not report the activity we observe in the startbox. The task relies on memory access by rats, which has been extensively demonstrated in the literature (to name a few, Day and Morris Science 2003, Wang et al PNAS 2010, Takeuchi et al Science 2011, Nonaka et al EJN 2017, Broadbent et al EJN 2020, Tse et al PNAS 2023). It is therefore a legitimate experimental question to observe the hippocampal dynamics in the rats used in this study in the time window where animals are likely to retrieve memories and plan their actions accordingly. The observed effects on the optogenetic inactivation of hippocampal activity in the same time window – where such neurons reactivations are observed – further supports this point. Similarly, it would be a disservice to reader to not acknowledge – unfortunately limited in this by space, but discussed at length elsewhere e.g. the Hippocampus Book 2nd Ed. Chapter 14) – the work by several groups providing evidence on various form of neural activity supporting or not future planning, including replay (e.g. Gillespie et al Neuron 2021, Widloski and Foster Neuron 2021). Nevertheless, we do appreciate the need for clarification of our intents and contextualization of our findings with the existing literature, including that addressing the role of replay in memory planning, and we have modified our text to avoid misinterpretations to clarify the differences between replay – intended as the sequential reactivation of cells measured by means of electrophysiology – and what our data show. We have now revised our Introduction to remove some of the confusion between hippocampal phenomena and correctly framed our data in the context of existing literature. Furthermore, we have addressed this in the Discussion.

2- The method employed to assess hippocampal “reactivation”, used to support some of the main claims of the paper (Fig 3-4) is not adequate. The authors should use some of the well well-established methods in the field, which are based on performing careful statistical testing of the significance of the temporal structure of neural activity during offline periods compared to neural patterns during behavior in the maze. Instead, they simply counted which cells were active in 10 s periods before trial start. This measure was them compared with manual (?) annotations of whether those cells were active when rats were exploring different sandwells. This approach is very far from current standards in the field and prevents any meaningful interpretation of the results of hippocampal activity during inter-trial periods. Among the many caveats, the approach does not consider the temporal structure of activity, it is biased by baseline activity levels of different cells, does not control for differences in brain state that correspond to very different network activity (e.g., theta oscillations and ripples), is not validated by proper statistical and shuffling methods.

We thank the Reviewer for the comment and have now added additional analyses to support our observation. In particular, we have confirmed our annotations with an automatic classification of cells, and controlled our findings for baseline activity. Our data suggests that the observed results are not due to intrinsic differences in baseline cell activity, or skewed representation of one or more of the sandwells. Furthermore, we have trained a multinomial logistic regression classifier and a naïve Bayesian decoder using well-separated training and test sets. The decoders were trained using the neural activity for each rat during Exploration, representing space in a pre-reward situation, and considered only moving points to avoid potential contamination from reactivation events during offline or resting-periods periods. This was then applied to the 10s before trial starts confirming a significant overrepresentation of sandwells compared to chance, confirming our results showing reactivation of multiple sandwells, which we interpret as evidence of hippocampal constructive activity in an imagination-like way, as also argued by others (e.g. Comrie et al Phil. Trans. R. Soc. B 2022). As discussed previously, we are aware that the limitation of one-photon calcium imaging in terms of sensitivity and temporal resolution prevents us from correlating the observed calcium signals with phenomena that can only be detected by means of electrophysiology such as ripples or theta oscillations. This point has been addressed in the Discussion.

3- The optogenetic manipulation results are useful and interesting. While the differential behavioral impact on allocentric and egocentric conditions is valuable, its underlying mechanisms are not possible to determine with the data presented. Furthermore, such prolonged silencing (3 min) is very problematic for multiple reasons. Among them, it silences different types of activity patterns (ripples as highlighted by the authors, but also theta oscillations and others), it allows time for effects to propagate through polysynaptic circuits beyond the hippocampus, can lead to cell accommodation and tissue over-heating. These caveats prevent a mechanistic interpretation of the behavioral effects. A better approach, nowadays common in the field, would be to transiently silence the hippocampus during specific activity patterns (such as ripples) or at least during specific discrete behavioral periods (perhaps harder in the context of this task).

In pilot animals, we tested the ability to silence neurons by means of optogenetic activation of JAWS opsin. Because our experiment lets rat have agency as to when exactly to exit the startboxes and start the trial, the illumination time will vary case by case as it will terminate when animals exit the startbox (sessions 27, 29), or reach the rewarded sandwell (sessions 31, 33). For this reason, we used 3 minutes as a reference interval where we could evaluate the effectiveness of neuron silencing. From our electrophysiology experiments, we can therefore safely conclude that we can achieve robust silencing for at least 3 minutes. We have now measured the effective illumination time in animals in the experiment and provided the data in new Supplementary Figure S36. As can be seen, the effective illumination does not in fact exceed 100s in most cases and in fact averages 30-40s in sessions 27,29 , and 40-60s in sessions 31,33. Closed-loop optogenetic stimulation during specific electrical activity, albeit feasible, are technically demanding and not exactly common in the literature. Even so, it has been reported that reactivations are not limited to SWRs (Widlowski and Foster Neuron 2022). Furthermore, our stimulation is in fact largely restricted to planning behaviour, since the range of behaviours in the small startboxes are quite limited (see also Gobbo et al PNAS 2022). Nevertheless, the Reviewer correctly notices that multiple activity patterns will likely be

silenced besides neuron firing and SWRs reduction; we have therefore raised the issue in the Discussion.

We wish to thank the Reviewers for the careful read of our paper and highlighting the novelty and interesting points. We have now revised the text according to their feedback. Reviewers will find the new text highlighted in blue and our responses to their individual points in blue below.

Reviewer #1 (Remarks to the Author):

I thank the authors for their thoughtful and extensive revisions, which have substantially improved the manuscript. The additional analyses, hierarchical bootstrap controls, decoder validation, and new figures documenting example Ca^{2+} traces and cell counts all strengthen the methodological rigor. The clarifications on place-field detection parameters, the reorganization of the text, and the updated discussion are also appreciated.

We thank the Reviewer for appreciating the substantial work and additional analyses prompted by theirs and the other Reviewers' comments.

That said, several conceptual issues remain only partially resolved and would benefit from further clarification.

1. Nature of "reactivation" and temporal limitations of Ca^{2+} imaging: The authors now refrain from using the term replay, which is a helpful step. However, the interpretation of the pre-trial hippocampal activity still remains conceptually difficult. Even with decoders and automatic cell classification, the underlying measure is a coarse quantification of which neurons were active during a 10 s period—not a temporal sequence, and not an event with defined network structure. I appreciate the authors' transparent recognition of this limitation, but the interpretation still appears stronger than what these signals can support. A clearer distinction is still needed between sequential "replay" phenomena, which require millisecond precision and fast-time structured patterns, and reactivation of coarsely overlapping populations, which is what can be extracted from 1-photon Ca^{2+} imaging.The authors need further sharpen the conceptual boundary in the Introduction and Discussion so that readers do not infer a level of temporal structure that cannot be resolved with the current method.

In the paper, our main intention was to understand how the navigational strategy impacted the representation of space as well as access to this information during decision making. We agree with the Reviewer that in the field a necessary clarification of terminology is necessary to avoid conflicting interpretations and clearly identify what biological phenomenon is at play. In our Revised text, we have followed the advice of the

Reviewers and of a consensus of experts in the field of memory reactivation/replay (Genzel et al Phil Trans Royal Soc B 2020). While we think that the term “reactivation” could be extended to our observation, we now avoided it for clarity and instead spoken of “neuronal activity” and ” neuronal activation”. We stress that it is not our intention to portray our data as replay instances and we are in agreement with the Reviewer about the temporal limitations of calcium imaging as a technique – chosen in our study specifically to allow longitudinal comparisons. To make this clear, we have removed references to “replay” from the introduction, kept only references that discuss nonlocal activation, and made a clear distinction between our data and evidence for replay and other forms of reactivation in the Discussion. We think it’s necessary to mention replay experiments in the Discussion out of completeness given our interpretation of startbox neuronal activation as planning activity, but the distinction is now made abundantly clear. In the Results, we have only used the term “activity”. For completeness of information to the reader, we have also calculated the temporal distributions of events in the startbox in the new Supplementary Figures 28 and 29. Again, we have stressed the limited temporal resolution of calcium imaging and only used 0.5s intervals to calculate the temporal proximity of events.

2. Interpretation of optogenetic manipulations: The authors nicely document the illumination times and discuss SWR-independent forms of prospective activity. These additions help. Still, the core interpretive issue remains unresolved because prolonged inhibition inevitably affects multiple network states and perhaps extra-hippocampal circuits, making difficult to attribute the behavioral effect to suppression of a specific hippocampal computation in the pre-startbox window. The manuscript would be strengthened by explicitly framing the optogenetic results as a state-dependent perturbation of hippocampal activity during the planning window, rather than as evidence for a mechanism tied to a specific physiological event class (e.g., SWRs).

We appreciate the insight and have changed the text accordingly. We have calculated the effect of illumination on SWRs not because we wish to link our observation to SWRs directly, but because, as the Reviewer points out, multiple effects might be at play, and we thought it was important to provide all the information we have available to the Reader.

3. Regarding my earlier point on potential laminar biases: I appreciate the authors’ expanded discussion on deep vs. superficial CA1 pyramidal cell differences. However,the revised text relies primarily on ephys studies, which given its millisecond temporal resolution captures dynamics that are not directly comparable to those measured with one-photon calcium imaging. Because the present study interprets differences in place coding and reactivation using calcium signals, it would be more appropriate to place the results in the context of imaging-based studies that have

quantified depth-dependent sampling biases and their impact on spatial tuning and stability (e.g., PMID: 27397517; PMID: 40015277). Incorporating these imaging perspectives would clarify how potential laminar biases may influence the results and would make the discussion more internally consistent with the methodological framework of the paper.

We thank the Reviewer for pointing this out and we have included the suggested work in the Discussion. We also clarified, for each experiment, whether the data were obtained in electrophysiology or calcium imaging experiments.

Reviewer #1 (Remarks on code availability):

n/a

Reviewer #2 (Remarks to the Author):

The revision has largely addressed concerns, and particularly the discussion now appropriately discusses the limitations of the work. I would recommend including a block diagram (or diagrams) that summarizes all the experimental details related to what is tested at different sessions, and in Figure 1c,d, mark the 180-degree rotation trials. It is also curucial to mention that after session 19, the rotation trials were performed in curtain open (I assume) which is a cruicial detial, which i asked about in my previous review.

We thank the Reviewer for recognising the work done in our previous revision. We appreciate the suggestion and have included a block of diagram in Supplementary Figure S1. Complete trial-by-trial information is also present in Supplementary Tables 1 and 2. Of course, the Reviewer is correct in interpreting that rotation trials in Session 19 (and 32) were performed with cues available, and curtains open. We have now clarified this aspect of the protocol in the manuscript in the text and Supplementary Figure 1.

Reviewer #3 (Remarks to the Author):

In this revision, the authors have included a few new analysis and statistical controls as well as textual additions to the Introduction and Discussion. While I appreciate the new additions and, more so, the extensive amount of data already contained in this manuscript, the concerns I raised in my original revision still remain largely unsolved. I

will not ask for additional experiments or analyses, since this paper is already massive. Instead, I will reiterate below the major concerns with the framing of this study, interpretation of the results and general analytical framework that, unfortunately, still prevent me to endorse its publication.

One of my main initial concerns was the framing of the current study and its finding in relation to hippocampal "replay" or "reactivation". These are very well-established concepts in the field that refer to the "offline" recurrence of neural population activity patterns (either sequences in the case of replay or more generally ensemble cofiring in the case of reactivation). The two defining features of these physiological events are 1) they involve coordinated population activity, and 2) they rely on the preservation of the temporal structure of such activity but in a compressed manner compared to its occurrence during behavior. None of these features are present in the events and phenomena study in this work. With this, I do not want to imply that there is no value in the present work or in the observations reported by the authors. However, the current framing is very misleading and, in my view, the interpretation of the data is not accurate, and at odds with well-established standards in the field.

Despite my insistence on this same issue in my original revision, the only thing the authors have done to address it is to add some text in Introduction and Discussion to describe replay and other phenomena in the literature. While this is useful, it misses the main point which was reframing their interpretation. I will provide just a few examples to illustrate this point, but there are many more in the manuscript.

- The first sentence of the abstract still frames the problem relating it to the concept of replay: "It still remains unclear how the brain replays stored neural information…"

We thank the Reviewer for the feedback and recognising the large amount of work and effort spent on the manuscript. Overall, we agree with the Reviewer that it may be misleading to frame our work and our results in terms of replay, and this is not our intention. Where we have referenced replay it is not to present our results as patterns or sequences, but rather to relate them to existing evidence and prior literature on nonlocal activation in the form of replay, or relating it to theta sequences, and overall existing literature addressing internally generated representations for goal identification and/or action planning. Our central question and the framework of our study concern how navigational strategy influences spatial representation in the hippocampus and hippocampal dependency, both in the arena and in the startbox, which we reiterated in the Discussion *("The two key findings of this study are that (i) in startboxes at the edge of a training arena, hippocampal representations of remote locations, as captured in Ca2+ imaging, are different in allocentric-trained animals than in those egocentric-trained, and (ii) inhibition of this neural activity in the hippocampus can cause measurable and significant errors in the navigational choice performance of allocentric-trained animals.")*. We have therefore reworked the text to clarify this, and differentiated unequivocally our results, and approach, from the biological phenomenon of replay. We

have therefore privileged the term ‘activity’ and ‘events’ for the events detected in the startbox because that is what they are: neuronal activity and calcium events. We believe that this clears any confusion, addresses the points raised by the Reviewers, and is in line with the guidelines recently expressed by a consensus of experts in the field (Genzel et al 2020): “*one should return to primarily using terminology and descriptions that incorporate information about the technique used instead of inferring function, especially in short descriptions such as the abstract*”. In the revised manuscript we avoided any term related to replay when referring to our data, in particular in the revised the title, abstract, and results section.

- The authors keep insisting that reactivation is an appropriated term to describe their data and that it does not pose any confound with prior literature. As a proof that this is not the case, they themselves use this same term to refer to their observed phenomena (essential the firing of individual place cells encoding specific maze locations during inter-trial intervals in the start box) with the replay of compressed place cell sequences or the reactivation of cell assemblies (e.g., lines 77-85, lines 567-573). I want to stress that this framework is very misleading. Unfortunately, the new text added to the revised manuscript sometimes increases the confusion because it compares the observation of the authors with other types of events previously studied (replay, assemblies or theta sequences) that are completely different.

We apologize if we failed to sufficiently clarify our position. We have now removed references to replay and reactivation from the Introduction, and thoroughly reworked the Discussion. When referring to our results we now exclusively use the term 'non-local activity'. Further to the Reviewer’s point, we initially adopted the term reactivation in line with Genzel et al. who define: “*Reactivation: patterns present during learning/encoding are activated again more strongly at a later time point than prior to learning/encoding. The pattern does not have to be measured on the individual neuron level but instead can be present in any measuring technique (e.g. by measuring individual unit activity with intracranial recordings as well as multi-voxel pattern analysis in fMRI BOLD signal) or a variety of analysis methods*“. However, to avoid confusion we have now adopted the less connotated term of “activity”. We believe that this does not detract from our overall interpretation (activity used for planning), since animals at the time of recording are proficient and well-familiarised with the environment (each group trained to use either an allocentric or an egocentric navigational strategy) and therefore neurons active in the startbox represent spatial information or knowledge to the animal.

A major shortcoming of the study is that the analytical framework is based in the analysis of the activation of single neurons, rather than in the temporal structure of population activity. While that is something the field has already moved pass from, if the

authors still want to focus on this approach is wrong in itself. However, this should be clearly stated (currently it is not clearly evident) the interpretation of the data should be changed accordingly, and the comparison with prior studies of population patterns should be carefully made (currently it is not). For example, the paper (starting from the paper) keeps referring to the reactivation of planned spatial trajectories. This is far from accurate because reactivation of trajectories implies by definition a sequence of place cell. Instead, what the authors show is the "reactivation" of specific locations in the maze, not trajectories towards them. Again, the current framing and interpretation does a great disservice to the present work, which includes many interesting observations, and precludes an accurate understanding by readers.

As pointed out here and in our previous revision, it would not be appropriate for us to speak of sequences due to the temporal limitation of interring calcium events - which, on the other hand, allowed us to follow the same neurons across consecutive days – so that will have to remain a limitation of the study. We have addressed this in the Discussion unequivocally (third paragraph in the Discussion section), and, as noted above, distinguished our results from instances of reactivations that imply temporal structure. For the sake of completeness and transparency, we have now calculated the temporal clustering of events in the startbox – using a 0.5s time window – (Supplementary Figure 28,29) again underlying the difference between the time scale observed in the present manuscript and the one typical of replay and reactivation events. Last, we agree with the need to avoid the term "trajectories", which could imply the observation of sequences, and instead talk about activation of cells representing, or mapping, to individual locations of goals.

We thank the Reviewer for providing a critical and complementary view of our data, recognising where our work was of value according to their opinion. We believe that the manuscript is now stronger and less prone to unintended misinterpretation.

The new analysis included, while very limited in scope, are helpful. It was not easy for me to track the new analysis and figures since very little of them has been added to the main figures and text, and nothing is specifically labelled in the supplemental material. Overall it seems that the new analytical addition have been limited to provide some restricted controls while the main analysis framework, which was the object of my initial criticism, remains unchanged.

We apologise for the extra effort needed to identify the new added portions of text in the supplementary material, but the Submission system did not allow us to load two separate versions with and without highlighted changes.

Reviewer #3 (Remarks on code availability):

NA
